# Caffeic Acid Modulates Protein Disulfide Isomerase-NLRP3 Inflammasome Signaling to Mitigate Inflammation in Acute Pneumonia

**DOI:** 10.7150/ijbs.101061

**Published:** 2026-03-09

**Authors:** Guanjun Li, Tong Yang, Ying Zhang, Ang Ma, Lirun Zhou, Chen Wang, Peng Gao, Cui Liu, Junzhe Zhang, Yin Hua Zhu, Huan Tang, Jigang Wang

**Affiliations:** 1 State Key Laboratory for Quality Ensurance and Sustainable Use of Dao-di Herbs, Artemisinin Research Center, and Institute of Chinese Materia Medica, China Academy of Chinese Medical Sciences, Beijing 100700, China.; 2 State Key Laboratory of Veterinary Public Health and Safety, College of Veterinary Medicine, China Agricultural University, Beijing 100193, China.; 3 Department of Urology, Shenzhen Clinical Research Centre for Geriatrics, Shenzhen People's Hospital; The First Affiliated Hospital, Southern University of Science and Technology, Shenzhen 518020, Guangdong, China.; 4 Beijing Advanced Innovation Center for Food Nutrition and Human Health, Department of Nutrition and Health, China Agricultural University, Beijing 100193, China.; 5 State Key Laboratory of Antiviral Drugs, School of Pharmacy, Henan University, Kaifeng, China.

**Keywords:** Natural products and herbs, Caffeic acid, Anti-inflammation activity, Activity-based chemical proteomics, Protein disulfide isomerase

## Abstract

Caffeic acid (CA) is a polyphenol found in various of plants and daily beverages including coffee. It possesses diverse biological effects, including anti-inflammatory properties. Acute pneumonia represents a widespread inflammatory process; however, whether CA can mitigate acute pneumonia and its specific molecular mechanisms remain elusive. Here, we have demonstrated the robust anti-inflammatory effect of CA both *in vivo* and *in vitro*. Additionally, protein disulfide isomerase (PDI) was identified as a potential target of CA via activity-based protein profiling strategy coupled with a chemical probe of CA. Moreover, CA was found to covalently bind to PDI through cysteine sites. Subsequent *in vivo* and* in vitro* experiments further revealed the inhibition of PDI-mediated NLRP3 inflammasome signaling constituted the specific mechanism through which CA exerts anti-inflammatory effect. In conclusion, our study elucidates the molecular mechanisms underlying the amelioration of acute pneumonia by CA, providing valuable insights into its potential therapeutic application for inflammation-related diseases.

## Introduction

Characterized by pathogen infection of the respiratory tract, acute pneumonia represents a severe inflammatory disease of the respiratory system wherein lung inflammation is instigated by pathogens or other factors 1, 2. Mounting evidence underscores the indispensable role of immune cells and the cytokines they secrete in the development of acute pneumonia 3. Corticosteroids, such as dexamethasone, are widely used in the clinical management of acute pneumonia 4. Nonetheless, these drugs may induce a myriad of adverse side effects, such as a reduction in immune function and an increased risk of infections 5. Hence, it becomes imperative to explore potential therapeutic agents with fewer side effects and enhanced efficacy. A comprehensive understanding of the underlying mechanisms of promising alternative natural agents holds the potential to facilitate the development of effective treatment strategies aimed at alleviating the burden of acute pneumonia.

Caffeic acid (3,4-dihydroxycinnamic acid, CA) is a well-known phenolic compound widely distributed in the human diet, including coffee and tea 6. Extensive studies have shown that CA has pleiotropic pharmacological effects, including antioxidant, anti-tumor, immunomodulatory, cardio-protective, and anti-inflammatory effects 7. Furthermore, CA demonstrated to inhibit LPS-induced inflammation in primary bovine mammary epithelial cells and macrophages 8. However, the specific protein targets of CA's anti-inflammatory activity and the underlying molecular mechanisms through which CA ameliorates symptoms of acute pneumonia remain elusive.

Activity-based protein profiling (ABPP) is a chemical proteomic approach aimed at elucidating the interaction mechanisms between small bioactive molecules and protein targets 9. Over the past two decades, ABPP has emerged as a powerful tool for dissecting the modes of action of natural drugs 10. This method enables the investigation of the targetability and selectivity profile of drugs and natural products in living systems, offering the advantage of target identification and validation at the proteomic level 11. Therefore, we employed ABPP to elucidate the covalent binding targets and the detailed mechanism underlying the anti-inflammatory effects of CA in acute pneumonia.

In this study, we initially assessed the efficacy of CA in alleviating LPS-induced acute pneumonia using both *in vitro* and *in vivo* models. Subsequently, a chemical probe containing an alkyne tag (CA-P) was devised to identify potential protein targets in LPS-induced macrophages by employing ABPP. Through this approach, we highlighted the protein disulfide isomerase (PDI) protein family as functional covalent targets, elucidating the specific molecular mechanisms underlying CA's anti-inflammatory activity through covalent interactions. Our aim is to provide novel insights into the molecular mechanisms driving the effects of CA on acute pneumonia and inflammation-related disorders.

## Materials and Methods

### Animal experiments

Forty male Balb/c mice (8 weeks old) were obtained from Vital River Laboratory Animal Technology and randomly divided into 5 groups (n=8 per group): the control group, model group, DEX (positive drug) group (10 mg/kg), CA-Low dose group (10 mg/kg), and CA-High dose group (20 mg/kg). Acute pneumonia was induced by administering LPS (10 mg/kg) via tracheal intubation after anesthesia. Blood samples were collected from the mice's eye sockets and centrifuged at 3000 g for 5 min to obtain serum for subsequent analysis. Lung tissue was collected post-sacrifice and fixed in 4% paraformaldehyde (PFA) for subsequent experiments.

### Hematoxylin-eosin (HE) staining and immunohistochemistry

Lung tissue samples obtained from mice were first embedded in paraffin, fixed with a fixative for approximately 10 minutes, and then washed with PBS. Subsequently, the samples were stained with hematoxylin-eosin (HE) solution for 10 minutes. For immunohistochemistry analysis, tissue sections were incubated overnight at 4 °C with a TNF-α antibody (dilution 1:200). The next day, the samples were permeabilized with DAB and 3% H_2_O_2_ for 10 minutes. Finally, the sections were sealed with neutral gum and observed under a microscope to capture images.

### Cell culture

RAW 264.7 cells were cultured in DMEM medium supplemented with 10% FBS at 37 °C in a humidified atmosphere with 5% CO_2_. To induce inflammation, cells were treated with lipopolysaccharide (LPS) at a concentration of 1 μg/mL.

### Synthesis of CA-P

CA and alkynylamine (in a 1:1 molar ratio) were combined in N,N-dimethylformamide (DMF) solvent and condensed using the condensing agent 6-chlorobenzotriazole-1,1,3,3-tetramethyluronium hexafluorophosphate (HATU, in a 3:1 molar ratio to CA) and the alkaline reagent N,N-diisopropylethylamine (DIPEA, in a 6:1 molar ratio to CA). Following the reaction, water and the organic extractant ethyl acetate were sequentially added to the solution. The organic phases were then combined after multiple extractions, concentrated, and subjected to column chromatography to yield white crystals, identified as CA-Probe.

### Cell viability

RAW 264.7 cells were seeded in 96-well plates and treated with different concentrations of CA or CA-P. After 24 hours, the supernatant was removed, and 10% CCK-8 reagent was added to each well. The plates were then incubated at 37 °C for 3 hours to facilitate the reaction.

### Measurement of NO and inflammatory cytokines

RAW 264.7 cells were seeded in a 24-well plate and treated with either CA or CA-P. The release of nitric oxide (NO) and inflammatory factors, including TNF-α, IL-1β, and IL-6, in the cell supernatant was measured using corresponding kits (Biodee for NO, Biorigin for TNF-α, IL-1β, and IL-6).

### Western blot

The detailed experimental procedure is described in the previous section 12. RAW 264.7 cells were lysed in RIPA buffer solution (Beyotime, China). Proteins were separated using SDS-PAGE (10%) and subsequently transferred to polyvinylidene fluoride (PVDF) membranes (Millipore). After blocking with 5% BSA for 1 hour, the membranes were incubated with primary antibodies at 4 °C overnight. The membranes were washed with 1x TBST and then incubated with HRP-conjugated goat anti-rabbit or anti-mouse IgG for 1 hour. Western blot bands were visualized using a chemiluminescence imaging system (CLiNX). The following primary antibodies were used: rabbit-PDI (11245-1-AP), rabbit-NLRP3 (27458-1-AP), rabbit-ASC (10500-1-AP), rabbit-caspase-1 (22915-1-AP), and mouse anti-β-Actin (81115-1-RR) from Proteintech. Secondary antibodies included HRP-conjugated goat anti-rabbit (111-035-003) and goat anti-mouse (115-035-003) antibodies from Jackson Immuno Research.

### Labeling and competition experiment

RAW 264.7 cells were treated with DMSO or CA for 3 hours, followed by two washes with PBS, and then collected. For the competitive group, cells were pre-incubated with varying concentrations of CA for 3 hours, followed by incubation with CA-P for 1.5 hours, PBS washing, and subsequent collection. Cell lysates were quantified using the BCA method, and then subjected to click chemistry reaction with a click reagent solution containing 1 mmol/L NaVc, 100 mmol/L THPTA, 1 mmol/L CuSO_4_, and 50 mmol/L TAMRA-N_3_ for 1 hour. The labeled proteins were precipitated using pre-chilled acetone (-20 °C). Subsequently, the proteins were mixed with 1× loading buffer, and fluorescence imaging was conducted using a hypersensitive multi-imager. Protein visualization was further performed using Coomassie Brilliant Blue rapid stain.

### Preparation of samples for LC-MS/MS and pull-down experiments

LPS-induced RAW 264.7 cells were prepared and divided into three groups: Control group, probe group, and competition group. DMSO or CA was added to the cells and incubated for 3 h, followed by the addition of CA-P and incubation for 1.5 h. The cells were then washed with PBS, lysed with lysis solution, and quantified using a BCA agent. Subsequently, the samples were normalized to a total protein concentration of 1 mg each. Click reagent was added to the normalized samples and allowed to react for 1 h, followed by the addition of cold methanol to precipitate the proteins. The precipitated proteins were then dispersed in 10% SDS/PBS and heated at 95 °C for 10 minutes. The supernatant was incubated with commercial streptavidin beads at room temperature for 4 h. Afterward, the beads were washed sequentially with 1% SDS/PBS (twice), 0.1% SDS/PBS (twice), and 6M urea/PBS. DTT (10 mM) and IAA (20 mM) were added and allowed to react for 30 min in the dark. The beads were then re-dispersed in PBS containing 1 M urea, 1 mM CaCl_2_, 25 mM NH_4_HCO_3_, and 2 μg trypsin at 37 °C overnight. Next, desalting of the sample prior to loading was performed using a commercial C18 column. The sample was dried and reconstituted with TEAB, followed by the addition of different TMT reagents (TMT_10_-129N, TMT_10_-130N, TMT_10_-131) to each of the three groups for labeling. TMT_10_-130N label reagents was labeled for negative control samples; TMT_10_-131 was labeled for CA-probe-treated samples; TMT_10_-129N was labeled for CA-probe-treated samples in the presence of CA, respectively.

For the pull-down experiment, the enrichment reaction procedure was similar to that mentioned above. After the enrichment reaction, the beads were incubated with loading buffer for subsequent Western blotting experiments.

### LC-MS/MS and data analysis

The detailed experimental procedure is described in the preceding section 13. The solubilized peptides were analyzed using an ultiMate 3000 nano LC system coupled with an Orbitrap Fusion Lumos mass spectrometer. Peptides were initially loaded onto an Acclaim PepMap100 C18 Nano-Trap column (100 Å, 3 µm, 75 mm × 2 cm) using a buffer containing 0.5% formic acid and 2% acetonitrile at a flow rate of 5 µL/min for 5 min. Subsequently, the peptides were eluted using a gradient of eluents A (0.1% formic acid in water) and B (0.1% formic acid in 80% acetonitrile/water) on an Acclaim PepMap100 C18 HPLC reversed-phase analytical column (130 Å, 2 µm, 75 µm × 250 mm) over a period of 75 minutes at 40 °C. Mass spectrometry data were collected and analyzed using the Xcalibur analysis system and processed with PD V2.4 for database analysis.

### Immunofluorescence assay

RAW 264.7 cells were seeded in confocal dishes and stimulated with LPS. After LPS induction, cells were treated with CA for 3 h, followed by incubation with CA-P for an additional 1.5 h. Subsequently, the cells were washed three times with PBS and fixed with 4% paraformaldehyde (PFA) for 15 min. After fixation, cells were permeabilized with 0.2% Triton X-100 and blocked with 5% BSA for 1 h. Next, click reagent was added and allowed to react for 1 h, followed by overnight incubation at 4 °C with the primary antibody against PDI (1:500). The next day, the secondary antibody (1:500) was added and incubated at 37 °C for 1 h. Hoechst stain was then added and incubated for 30 min to visualize the cell nuclei. Finally, images were acquired using a confocal microscope (Leica TCS SP8 SR).

### Purification of recombinant PDI and mutant

The pET30a plasmids expressing wild-type (PDI) or mutant PDI (mut) were transformed into *E. coli* BL21 cells. Monoclonal colonies were selected and propagated by shaking at 25 °C. Subsequently, the cells were induced with IPTG at 17 °C overnight to express the recombinant proteins. The induced cells were then harvested, lysed, and centrifuged to obtain the supernatant containing the expressed proteins. The supernatant containing the His-tagged proteins was applied to a nickel column for affinity purification. The column was washed with a series of imidazole concentrations (10, 20, 50, 100, 150, and 200 mM) to elute nonspecifically bound proteins, while the His-tagged proteins remained bound to the column. After washing, the specifically bound His-tagged proteins were eluted from the column using a high concentration of imidazole. The eluate containing the purified recombinant PDI was then ultra-filtered and concentrated to obtain a pure protein sample.

### Probe labeling of recombinant PDI and CA competition experiment

The purified proteins were treated with CA for 3 hours at room temperature (RT) and with CA-P for 1.5 hours, followed by addition of the click chemistry reaction cocktail and incubation for 1 hour at RT. The proteins were then separated using SDS-PAGE according to the previously described protocol.

### Cellular thermal shift assay (CETSA)

CA or DMSO was added to LPS-induced RAW 264.7 cells and incubated for 1 h. Then, the cells were transferred to PCR tubes. The gradient PCR apparatus was set to eight temperatures ranging from 37 to 72 °C (37, 42, 47, 52, 57, 62, 67 and 72 °C). Samples were heated for 5 minutes and subsequently centrifuged for 10 minutes to collect the supernatant for subsequent WB detection. For the CETSA experiment, the aim is to test the thermal stabilization of PDI protein in the absence and presence of CA. If there is an interaction between PDI and CA, PDI protein incubated with CA should exhibit significantly higher thermal stabilization compared to the counterpart incubated with DMSO.

### Detection method of PDI redox state

As previously reported 14, purified PDI proteins were pretreated with 100 mM DTT. CA was then added simultaneously and allowed to react for 1 hour at room temperature (RT). Next, samples were incubated with or without PEG-5k for 30 minutes. Protein migration was assessed using 10% SDS-PAGE.

### Preparation of the binding site sample of PDI and CA

The recombinant PDI protein was incubated with CA at 4°C overnight. Afterwards, a solution of methanol: chloroform: water (4:3:1) was added to precipitate the protein. Following precipitation, the samples were reconstituted in EPPS solution and subjected to enzyme digestion. Commercial C18 columns were used for desalting, spin drying, and reloading.

### Knockdown (KD) of PDI *in vitro*

The siRNA sequence targeting PDI (5'-CGAGUUCACUGAACAGACA-3') was designed and synthesized by Sangon as previously described. The siRNA and vectors were diluted in Opti-MEM medium and transfected into RAW 264.7 cells using RNAiMAX according to the manufacturer's instructions. After transfection for 6 hours, the medium was replaced with a serum-containing medium.

### *In vivo* PDI KD

The target sequences were as follows: PDI-siRNA, 5'-CCUUUGCUAGCGAAUCUCAGAGCC-3'; a plasmid transfected with scramble siRNA (5'-TTCTCCGAACGTGTCACGT-3') served as a negative control (NC) and was synthesized by Genomeditech Co. Ltd in Shanghai, China. The selected oligonucleotides were then cloned into the AAV plasmid vector to construct the AAV-PDI knockdown (KD) vector provided by Genomeditech. The recombinant AAV vector (5×10^12^ virus genomes/mL), diluted in PBS, was administered intranasally in a volume of 500 nL. Experiments to confirm viral transfer were conducted 3 weeks after AAV injection. This was followed by LPS induction to establish an inflammatory model and subsequent treatment with CA, as described previously.

### Statistical analysis

The analysis of Western blot bands was conducted using Image J software. Data were expressed as the mean ± SEM for at least three independent experiments and were analyzed using one-way ANOVA and two-way ANOVA. Statistical analyses were performed using GraphPad Prism version 9.0.

## Results

### CA exhibits anti-inflammation effects in acute pneumonia mice

**Figure 1:**
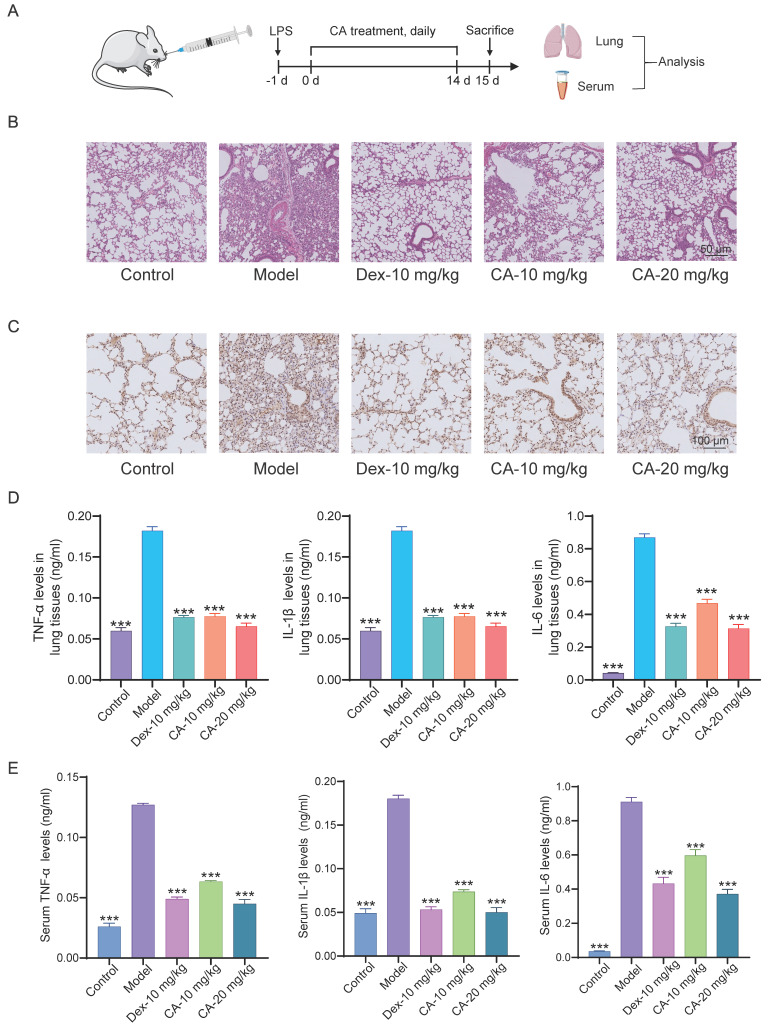
**Anti-inflammatory effect of CA in mice with acute pneumonia.** (A) Flow chart illustrating the process of animal modeling and drug delivery. (B) Representative photomicrographs displaying HE staining of lung tissues, scale = 50 μm. (C) Immunohistochemical results depicting TNF-α expression in mouse lung tissues, scale = 100 μm. (D) Quantification of inflammatory factors, including TNF-α, IL-1β, and IL-6, in lung tissues, n=8. (E) Analysis of inflammatory factor levels in serum, n=3. *** P<0.001 vs Model group. Control: DMSO treatment; Model: LPS-induced mice; DEX: dexamethasone (DEX) treatment (10 mg/kg); CA-Low: CA treatment (10 mg/kg); CA-High: CA treatment (20 mg/kg).

First, we investigated the anti-inflammatory effects of CA *in vivo* (**Figure 1**). Lipopolysaccharide (LPS), a major component of the outer membrane of gram-negative bacteria, stimulates the innate immune system, leading to inflammatory and toxic effects 15. Thus, we induced acute pneumonia in mice using LPS, followed by daily CA treatment for 14 days (**Figure 1A**). Histological examination of lung tissues confirmed that LPS induced significant lung damage, characterized by tissue injury and inflammatory infiltration in the model group. Dexamethasone (DEX), a classical corticosteroid 16, served as a positive control for anti-inflammatory activity. Furthermore, CA treatment dose-dependently attenuated these histological damages **(Figure 1B)**. Immunohistochemical analysis revealed that both CA and DEX alleviated the LPS-induced elevation of TNF-α (tumor necrosis factor) levels in mouse lung tissues **(Figure 1C)**. Additionally, analysis of inflammatory cytokines, including TNF-α, IL-1β (interleukin 1β), and IL-6 (interleukin6) in lung tissues of mice, showed a significant decrease in the levels of these cytokines in the CA-treated group compared to the model group **(Figure 1D)**. Moreover, consistent findings were observed in serum, where CA suppressed the elevated levels of TNF-α, IL-1β, and IL-6 in acute pneumonia mice (**Figure 1E**). Collectively, these data suggest that CA exhibits remarkable anti-inflammatory properties *in vivo*.

### CA and CA-P exhibit anti-inflammation effects in LPS-induced macrophage

**Figure 2:**
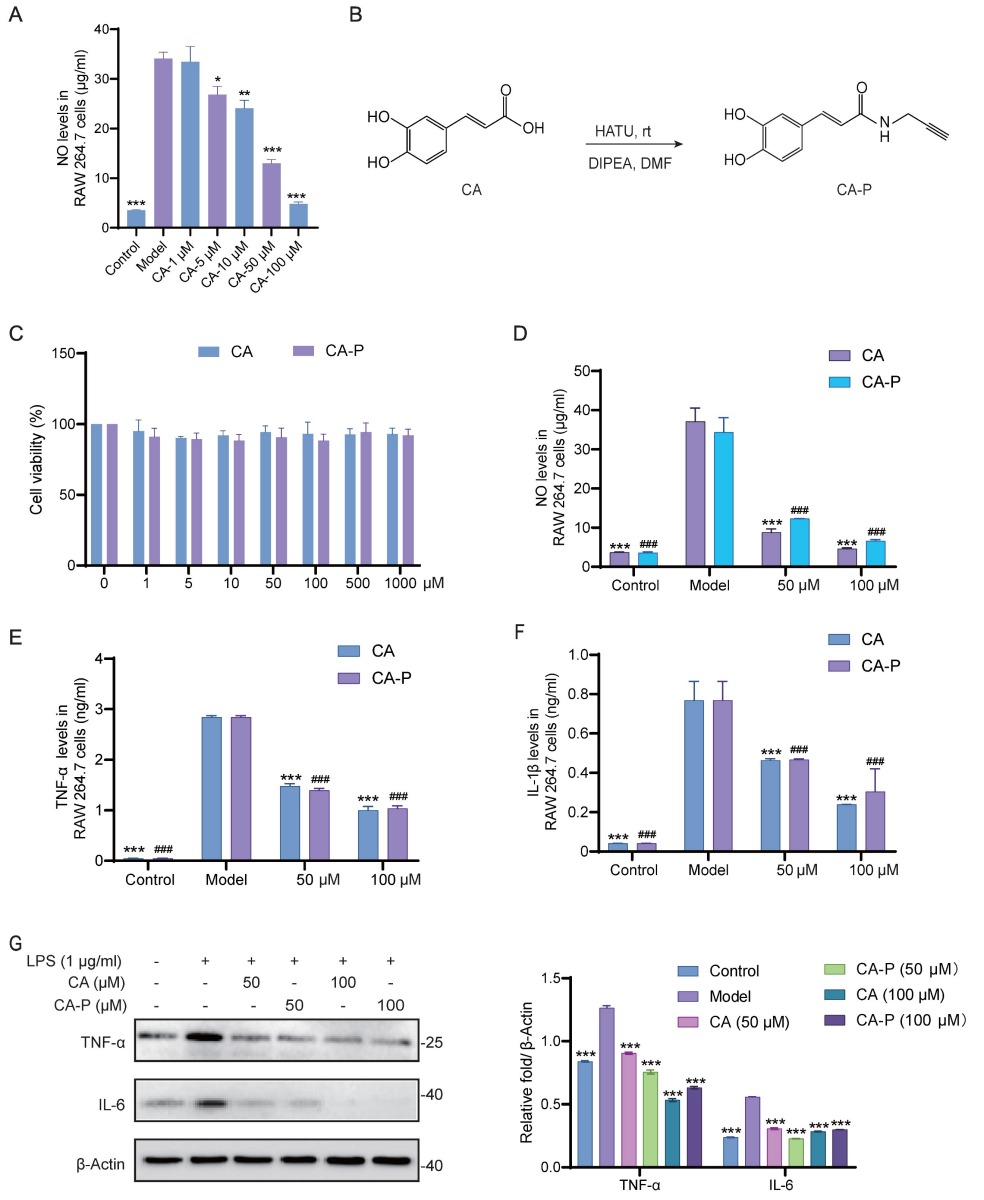
**Anti-inflammatory effect of CA and CA-P in LPS stimulated macrophages.** (A) NO levels in RAW 264.7 cells with CA, n=3, * P <0.05, ** P <0.01, *** P <0.001 vs model group. (B) Chemical structure and synthesis step of CA-P. (C) Cell viability of CA and CA-P in RAW 264.7 cells, n=3. (D-F) Levels of NO, TNF-α and IL-1β released from RAW 264.7 cells after treatment with CA or CA-P, n=3, *** P <0.001 in CA treatment group, ### P <0.001 in CA-P treatment group, compared to Model group. (G) Expression levels and corresponding densitometry of TNF-α and IL-6 in CA or CA-P treated RAW 264.7 cells, *** P <0.001, compared to Model group.

To validate and extend our *in vivo* findings, we utilized mouse macrophage cells (RAW 264.7 cells) as an *in vitro* model. As illustrated in **Figure 2A**, CA treatment significantly inhibited the levels of NO in LPS-induced RAW 264.7 cells in a dose-dependent manner, indicating the potent anti-inflammatory activity of CA. To profile the protein targets of CA responsible for its anti-inflammatory activity, we designed and successfully synthesized a chemical probe bearing an alkynyl group of CA (CA-P) (**Figure 2B** and **Figure S1**). Subsequently, we conducted experiments to verify whether CA-P and CA exhibited similar anti-inflammatory bioactivities. Firstly, we evaluated the cytotoxicity of CA and CA-P against RAW 264.7 cells using a CCK8 assay, and both CA-P and CA demonstrated biosafety up to 1 mM (**Figure 2C**). Additionally, the levels of NO and inflammatory cytokines in LPS-induced RAW 264.7 cells indicated that both CA and CA-P dose-dependently inhibited the release of inflammatory cytokines in cells, revealing that CA-P and CA possessed similar anti-inflammatory biological activities (**Figure 2D-F** and **Figure S2**). Furthermore, Western blot results showed that CA-P significantly decreased the expression levels of TNF-α and IL-6, similar to CA (**Figure 2G**). These results collectively demonstrate that both CA and its chemical probe possess potent and comparable anti-inflammatory activity *in vitro*, laying the groundwork for the subsequent target engagement by chemical proteomics.

### Profiling protein targets of CA in living macrophage by ABPP

**Figure 3:**
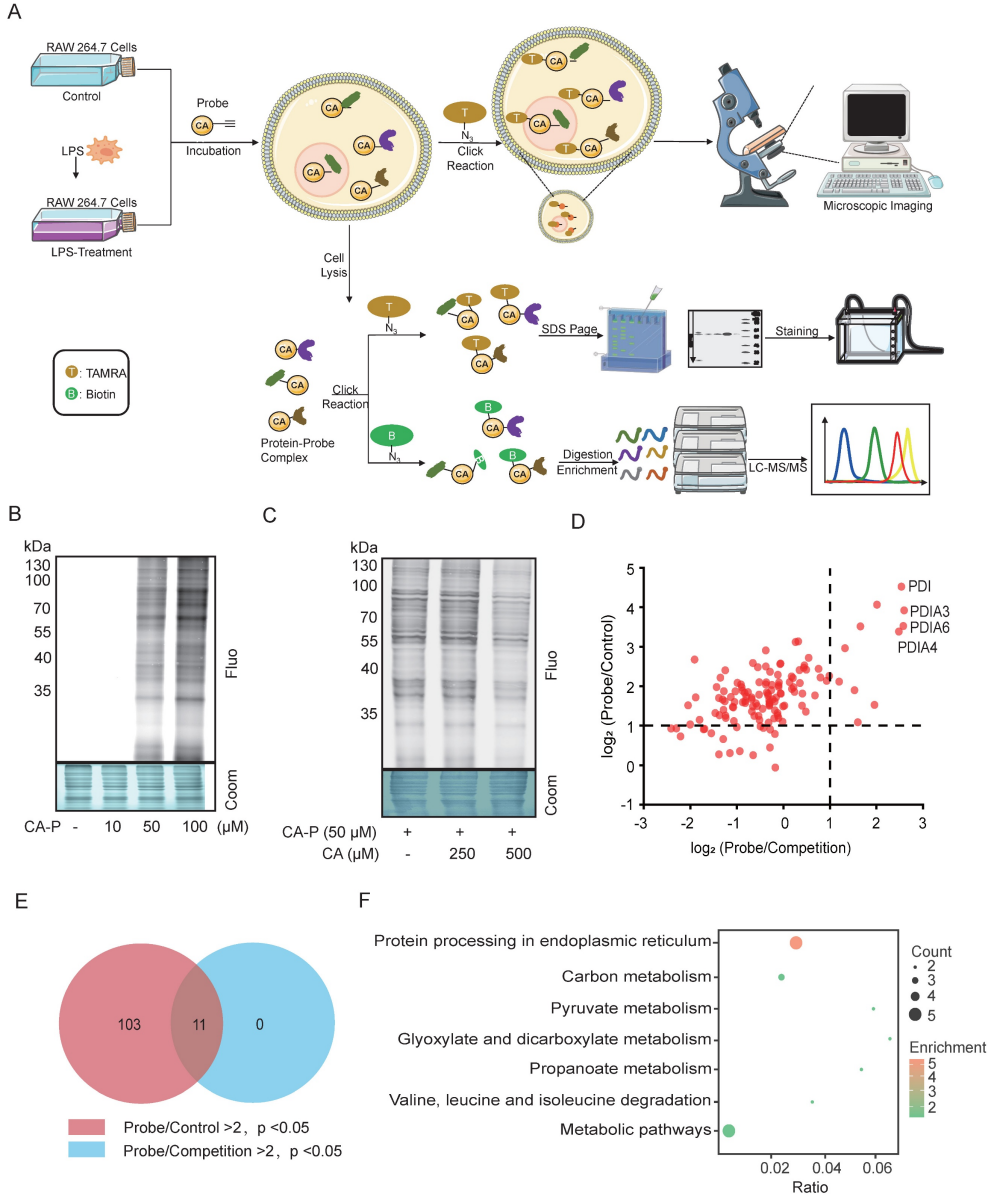
**Identification of CA-targeting proteins via ABPP approach in macrophages.** (A) Overall workflow of ABPP to identify CA-targeting proteins. (B) Fluorescence labelling of proteins captured by CA-P in the living cells. (C) Fluorescence labelling of proteins captured by CA-P (50 μM) in the living cells in the presence of CA (0; 250; 500 μM). (D) Scatter diagram of protein targets in the probe and competition groups. (E) Venn diagram showing the overlap of protein targets in the probe and competition groups. (F) KEGG analysis for overlapped targets.

Subsequently, we employed ABPP to identify the potential target proteins of CA in LPS-induced macrophages. As illustrated in **Figure 3A**, three groups were prepared as follows: the control group consisted of cells incubated with vehicles (DMSO); the probe group involved cells treated with CA-P; and the competition group comprised cells cultured with CA-P along with CA. Next, through the click reaction with rhodamine-azide and biotin-azide, the proteins captured by CA-P in LPS-treated RAW 264.7 cells were directly visualized and identified by SDS-PAGE and LC-MS/MS, respectively (**Figure 3A**). The fluorescence imaging of the gel showed that CA-P labeled proteins within the living cells, with intensity increasing with the concentrations (**Figure 3B**). Moreover, preincubation with excess CA efficiently competed away the labeled signal of CA-P on proteins in a dose-dependent manner, suggesting that CA-P shares protein targets with CA (**Figure 3C**). To further elucidate the identities of proteins targeted by CA, the proteins captured by CA-P were enriched by avidin beads and subjected to isobaric tandem mass tagging (TMT)-based LC-MS/MS analysis **(Table S1)**. The identified protein hits were depicted by corresponding volcano plots (**Figure S3**) and scatter plots (**Figure 3D**). Applying a cutoff ratio > 2 and a p-value < 0.05 in both the probe versus control group and the probe versus competition group, we identified 114 and 11 potential targets, respectively. Furthermore, the 11 proteins overlapped in the two settings, as indicated by the Venn diagram results (**Figure 3E**). Subsequently, these targets underwent Kyoto Encyclopedia of Genes and Genomes (KEGG) analysis, revealing seven enriched signaling pathways (**Figure 3F, Figure S4**). Notably, protein processing in the endoplasmic reticulum stood out, primarily due to the involvement of several proteins from the protein-disulfide isomerase (PDI) family, namely PDIA4, PDIA3, and PDIA6. The PDI family plays a significant role in protein synthesis and maintaining cell structure and function. It is also associated with endoplasmic reticulum stress and inflammation 17, 18. Moreover, PDI, ranking top in fold change in both the probe/control and probe/competition groups, has been reported to regulate the NF-κB pathway through knockdown assays 19, 20. Therefore, we selected the PDI protein as the functional target to elucidate the mechanism of CA's anti-inflammatory activities.

### PDI is the covalent target of CA in LPS-induced RAW264.7 cells

**Figure 4:**
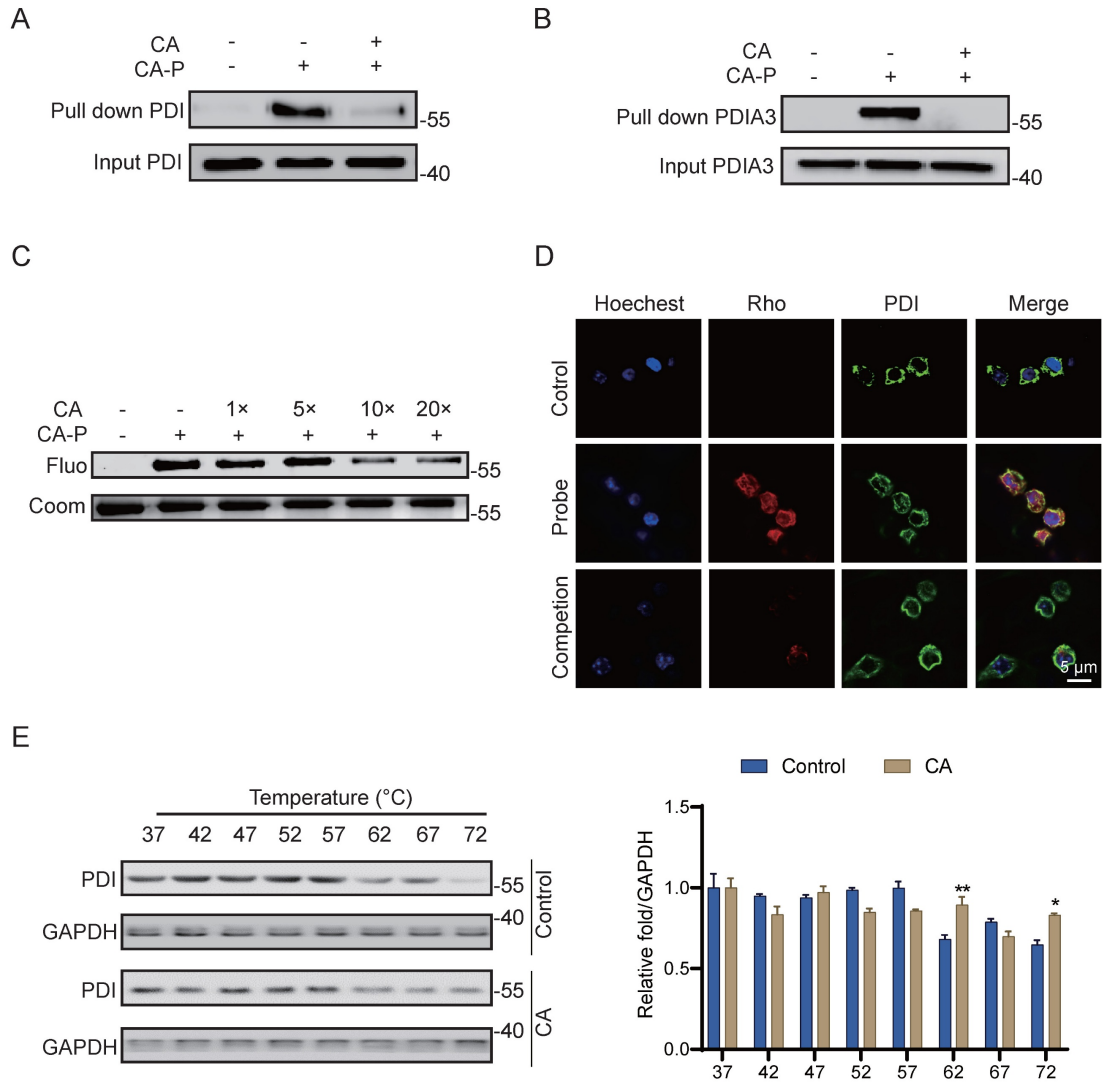
**PDI is the covalent target of CA.** (A-B) Pulldown assay of PDI and PDI3A by CA-P in cells. (C) Recombinant PDI protein labeled by CA-P in the absence and presence of excessive CA. (D) Cellular immunofluorescence staining of PDI (green) and CA-P (red), scale=5 μm. (E) CETSA-WB and statistical analysis results of PDI protein levels with or without CA, n=3, ** P <0.01, *** P <0.001, Control vs CA.

To further validate PDI as the robust covalent target of CA in living cells, we employed CA-P for pull-down experiments followed by immunoblotting assays. As anticipated, CA-P successfully pulled down PDI, and excess CA effectively competed against this binding (**Figure 4A**), indicating the covalent binding of CA to PDI in cells. Additionally, another member of the protein disulfide isomerase family, PDIA3, was confirmed as a covalent target of CA through a similar pull-down assay (**Figure 4B**), thereby corroborating the high credibility of the LC-MS/MS analysis. Recombinant PDI proteins were further incubated with CA-P in the absence and presence of excess CA. Consistent with the results obtained in the living cell system, excess CA competitively displaced the labeling signal of CA-P on PDI in a dose-dependent manner (**Figure 4C**). Furthermore, immunofluorescence staining with antibodies against PDI and CA-P, tagged with a red marker, revealed co-localization of the PDI protein with CA-P, with the fluorescence of CA-P significantly diminished by CA competition (**Figure 4D**). Moreover, we employed the cellular thermal shift assay (CETSA) coupled with western blot (WB) detection, a widely used technique for indicating ligand binding of drugs to proteins. This assay is based on observing protein melting changes after various heating steps and quantifying the remaining soluble protein 21. We investigated whether CA could stabilize PDI in cell lysate samples subjected to temperatures ranging from 37 to 72 °C to further confirm the binding. Compared with the control group, the CA-treated group notably stabilized PDI and decreased its degradation with increasing temperatures, indicating that CA directly targets PDI and enhances the stability of the protein **(Figure 4E)**. In summary, these results strongly support the conclusion that PDI is a robust covalent target of CA in LPS-induced living RAW264.7 cells.

### Cysteine residue is the modification site of CA binding to PDI

**Figure 5:**
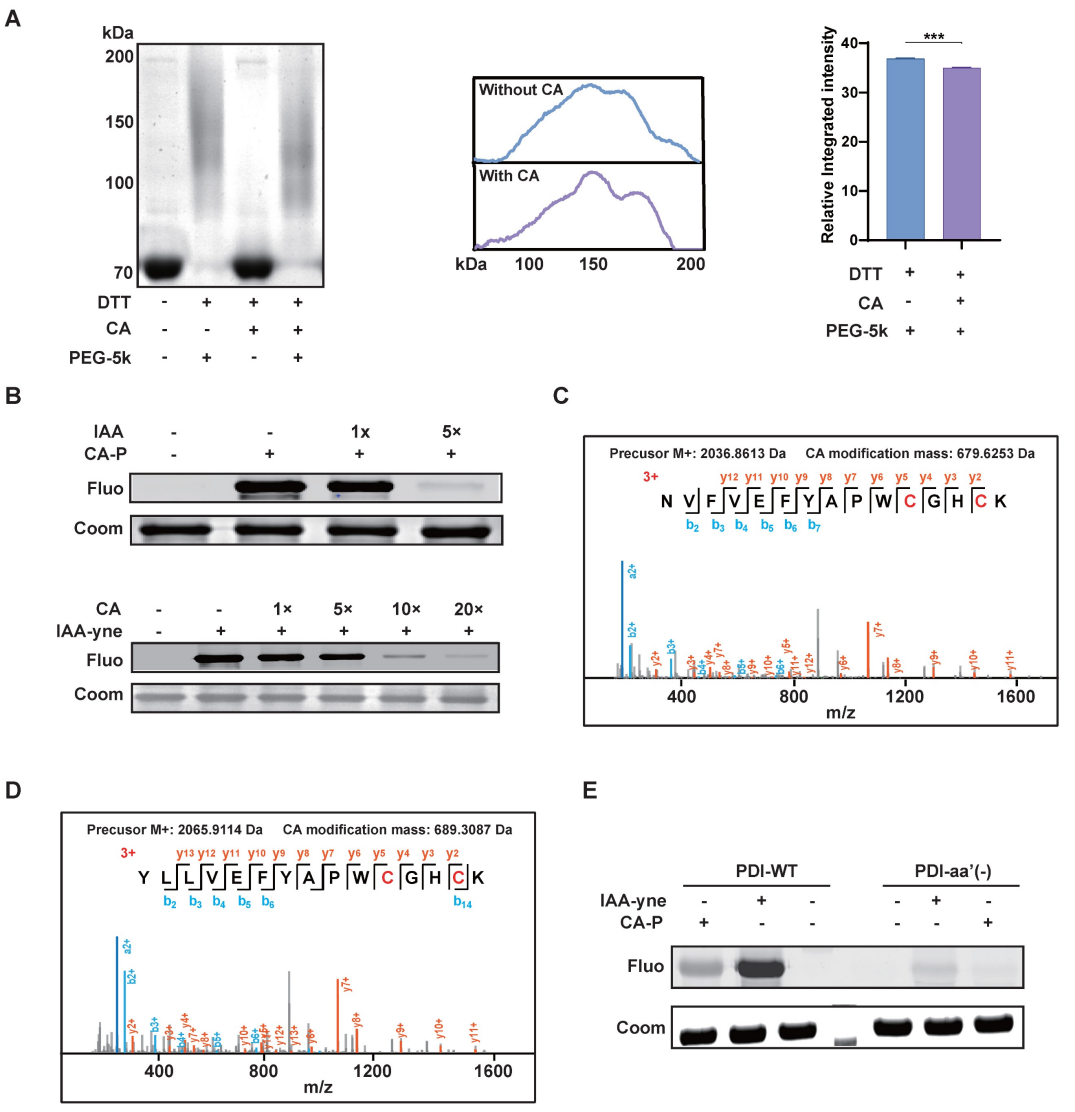
**Cysteine was the modification site of CA bound to PDI.** (A) Migration rate and intensity analysis of PDI labeled by PEG-5k in SDS-PAGE gel with or without CA treatment, n=3, *** P <0.001. (B) The competitive labeling of recombinant PDI protein with CA-P and IAA-P in the presence of excess IAA or CA. (C-D) MS2 analysis showing CA modified on cysteine sites of PDI. (E) The fluorescence labeling of PDI wildtype (PDI-WT) and PDI mutant (PDI-aa'(-)) with cysteine residues mutation by CA-P or IAA-P. IAA, iodoacetamide, an active alkylating reagent of cysteine; IAA-yne, a probe of IAA bearing alkynyl.

Subsequently, we conducted further experiments to elucidate the specific molecular functions of CA binding to PDI. PDI is intricately involved in the accurate synthesis of proteins through redox reactions and comprises four thioredoxin-reducing structural domains (a'-b'-b'-a'), with each domain containing two CGHC amino acid sequences, contributing to its enzyme activity 22, 23. The reducing agent dithiothreitol (DTT) has been shown to maintain PDI in a reduced state 24, and all four cysteines are susceptible to reaction with maleimide bearing methoxy polyethylene glycol 5000 (PEG-5k), resulting in the slowing down of protein migration in gel electrophoresis 14. Our results revealed that the migration rate increased following CA treatment **(Figure 5A)**, indicating that CA might inhibit the reductive activity of PDI via cysteine conjugation.

Given the pivotal role of cysteine residues in PDI and the presence of electrophilic α,β-unsaturated carbonyl groups in CA, which are prone to forming Michael addition reactions with cysteine residues 25, we investigated whether cysteine serves as the binding site of CA targeting PDI. To explore this, we subsequently found that iodoacetamide (IAA), an active alkylating reagent of cysteine, could block the binding of CA-P to the recombinant PDI protein. Conversely, CA significantly competed away the binding of IAA-yne (a probe of IAA bearing alkynyl) to PDI in a dose-dependent manner (**Figure 5B**). Furthermore, bottom-up proteomics analysis was conducted to identify CA modification sites on PDI. MS results revealed CA's covalent binding to all four cysteines (Cys55, Cys58, Cys399, and Cys402) within PDI's activity pocket (**Figure 5C-D**). Moreover, a mutated variant (PDI-aa'(-)) with targeted mutations of these four cysteines was generated, resulting in reduced labeling of both CA-P and IAA-yne (**Figure 5E**). Overall, these findings suggest CA specifically binds to PDI's four cysteines, and inhibited its reductive activity.

### CA exhibits anti-inflammation activity *in vitro* by inhibiting PDI mediated NLRP3 inflammasome

The NLRP3 inflammasome, comprising NLRP3 (NOD-like receptor family pyrin domain containing 3), ASC (apoptosis-associated speck-like protein containing a caspase-recruitment domain), and caspase-1, forms a multiprotein platform leading to caspase-1 activation, thereby regulating the maturation of pro-inflammatory cytokines such as interleukin-1β through direct cleavage 26, 27. Besides, it is reported that a PDI family protein is identified as a novel target for inhibiting NLRP3 inflammasome signaling 28. Hence, we hypothesized that CA binds to PDI and consequently inhibited the activation of NLRP3 inflammasome and exerted an anti-inflammatory effect *in vitro*.

**Figure 6:**
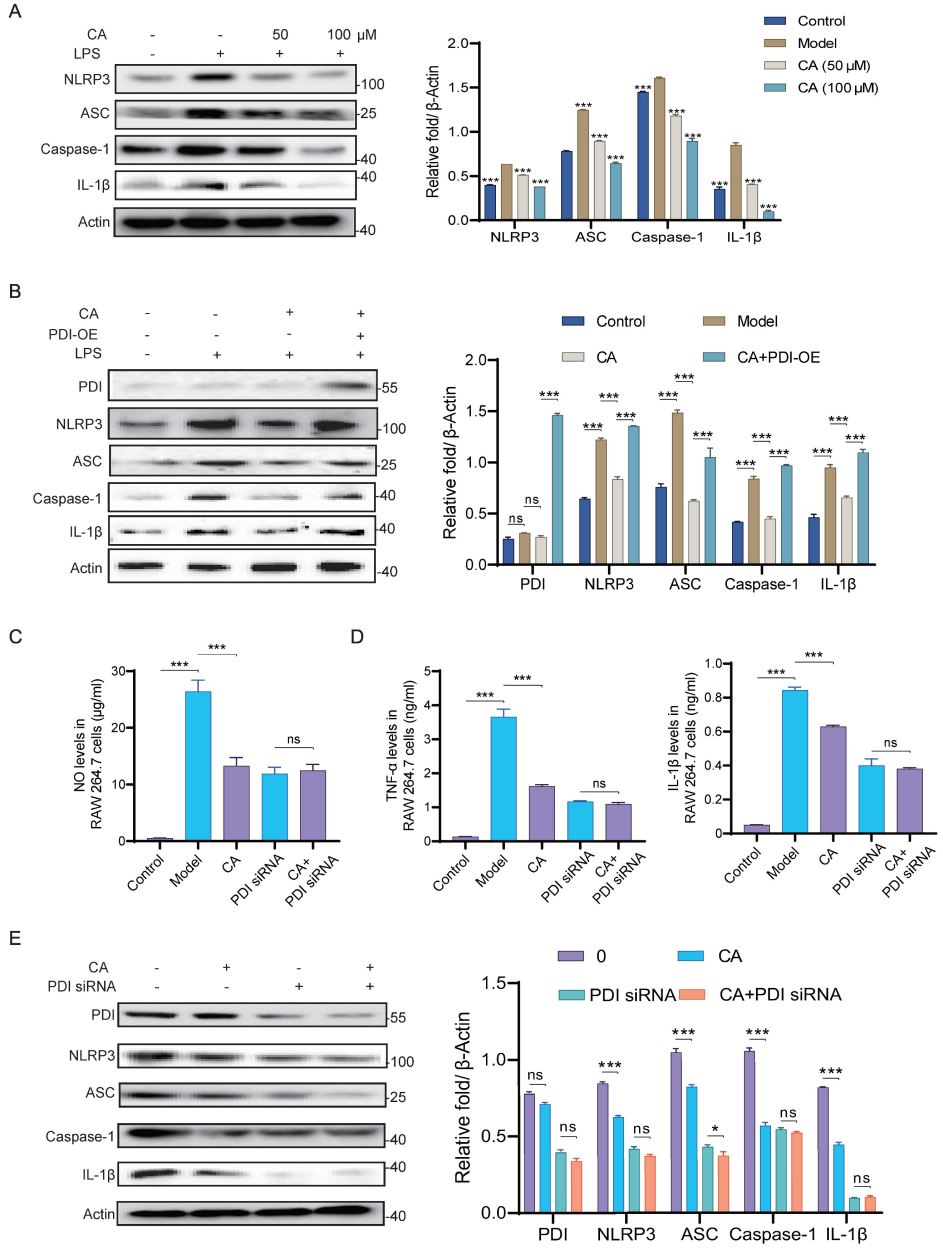
** CA inhibited inflammatory response via PDI mediated NLRP3 inflammasome pathway *in vitro*.** (A) Expression levels and statistical analysis of NLRP3 inflammasome proteins and IL-1β in RAW 264.7 cells treated with CA (50 or 100 μM) for 3 hours, n=3, *** P <0.001, compared with Model group. (B) Expression levels and statistical analyses of PDI and NLRP3 inflammasome proteins in RAW 264.7 cells treated with CA (50 μM) and PDI-OE. (C-D) The levels of NO, TNF-α, and IL-1β released from RAW 264.7 cells treated with CA and/or PDI siRNA. (E) Expression levels of PDI and NLRP3 inflammasome proteins in the LPS-induced RAW 264.7 cells treated with CA (50 μM) for 3 hours and/or PDI siRNA for 48 hours, and Image J soft was used to quantify the intensity. n=3 in B-F, ns, not significant, * P<0.05, *** P<0.001.

To further verify the mechanism by which CA exerts its anti-inflammatory effects via PDI, we assessed the protein levels of the NLRP3 inflammasome and IL-1β in LPS-induced RAW 264.7 cells. As illustrated in **Figure 6A**, CA suppressed the upregulation of NLRP3 inflammasome and IL-1β induced by LPS. Furthermore, overexpression of PDI restored the diminished expression levels of NLRP3 inflammasome and IL-1β induced by CA, indicating the requirement of PDI for CA to exert its anti-inflammatory effects (**Figure 6B**). Additionally, PDI siRNA nearly completely nullified CA's capability to reduce levels of NO and inflammatory factors (**Figure 6C-D**, **Figure S5**). In line with these observations, western blotting analysis demonstrated that CA markedly reduced the expression of PDI, NLRP3 inflammasome, and IL-1β, a reduction that was reversed by the addition of PDI siRNA (**Figure 6E**). In conclusion, these findings indicate that the anti-inflammatory effect of CA relies on PDI-mediated regulation of the NLRP3 inflammasome in RAW 264.7 cells.

### CA exhibits anti-inflammation effect via PDI mediated NLRP3 inflammasome *in vivo*

**Figure 7:**
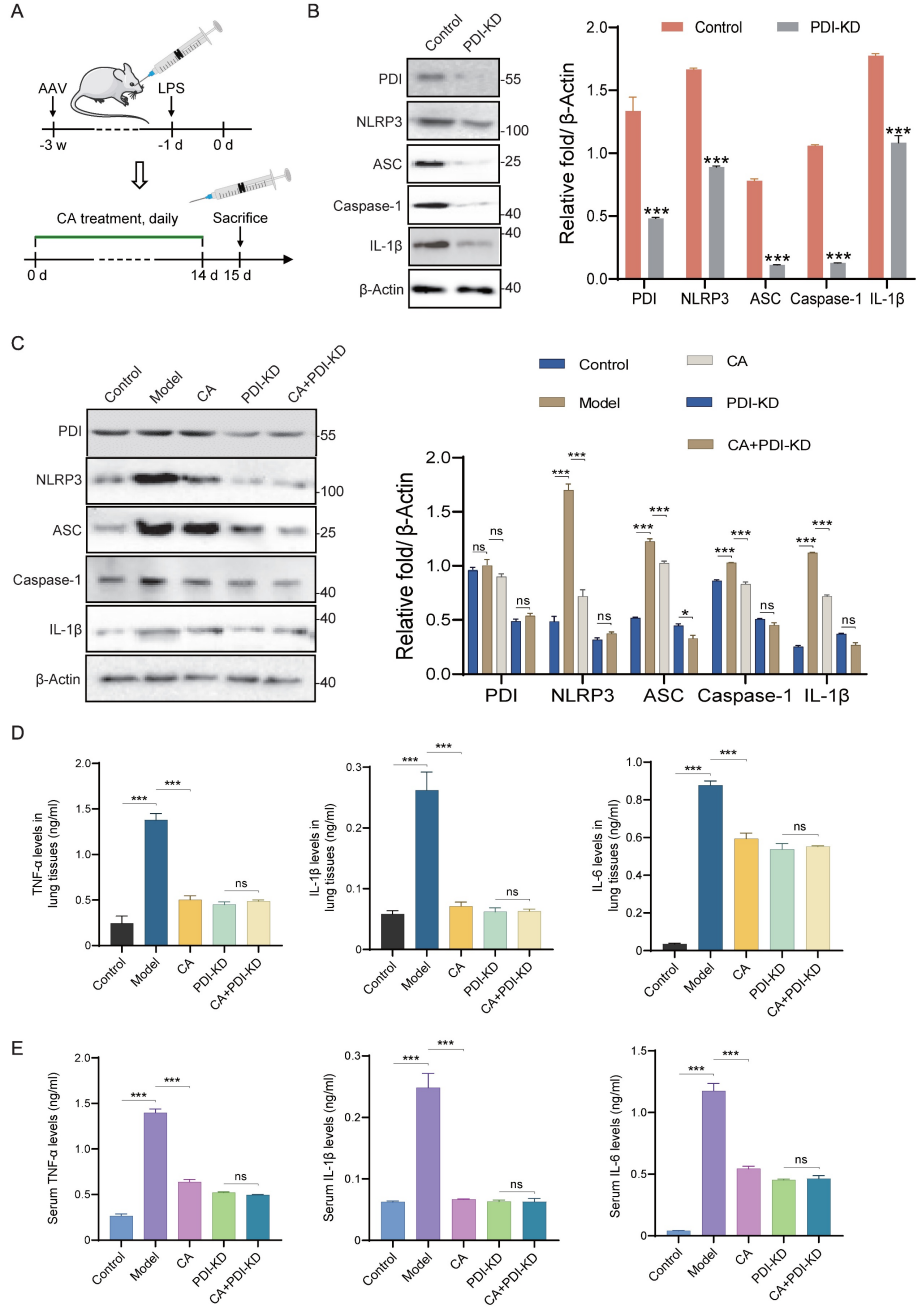
** CA inhibited inflammatory response via PDI mediated NLRP3 inflammasome pathway *in vivo*.** (A) Flow diagram of mice infected with PDI-KD AAV and treated with CA. (B) WB assay and densitometry analysis of proteins in lung tissues of mice with and without PDI-KD. (C) The expression levels of proteins in lung tissues of control or PDI-KD mice with CA treatment. (D-E) The levels of inflammatory cytokines in lung tissues (D) or serum (E) of PDI-KD mice after CA treatment. n=3, ns, not significant; *** P<0.001.

To further validate and extend our findings *in vivo*, PDI knockdown mice were generated, and inflammation was induced by administering LPS, followed by treatment with CA (**Figure 7A**). Western blotting analysis revealed significant knockdown of PDI, accompanied by a notable decrease in the expression levels of the NLRP3 inflammasome and IL-1β in lung tissues (**Figure 7B**). Furthermore, consistent with the *in vitro* studies, LPS administration increased the expressions of PDI, NLRP3 inflammasome, and inflammatory factors in mice, all of which were effectively suppressed by CA treatment. Importantly, this suppression mediated by CA was significantly attenuated in mice with PDI knockdown (**Figure 7C**). Correspondingly, ELISA analysis indicated that PDI knockdown nearly completely nullified CA's capability to reduce inflammatory factors in both lung tissues and serum (**Figure 7D-E**). In summary, these findings provide additional evidence that CA exerts its anti-inflammatory effects through PDI-mediated regulation of the NLRP3 inflammasome in mice.

## Discussion

**Figure 8:**
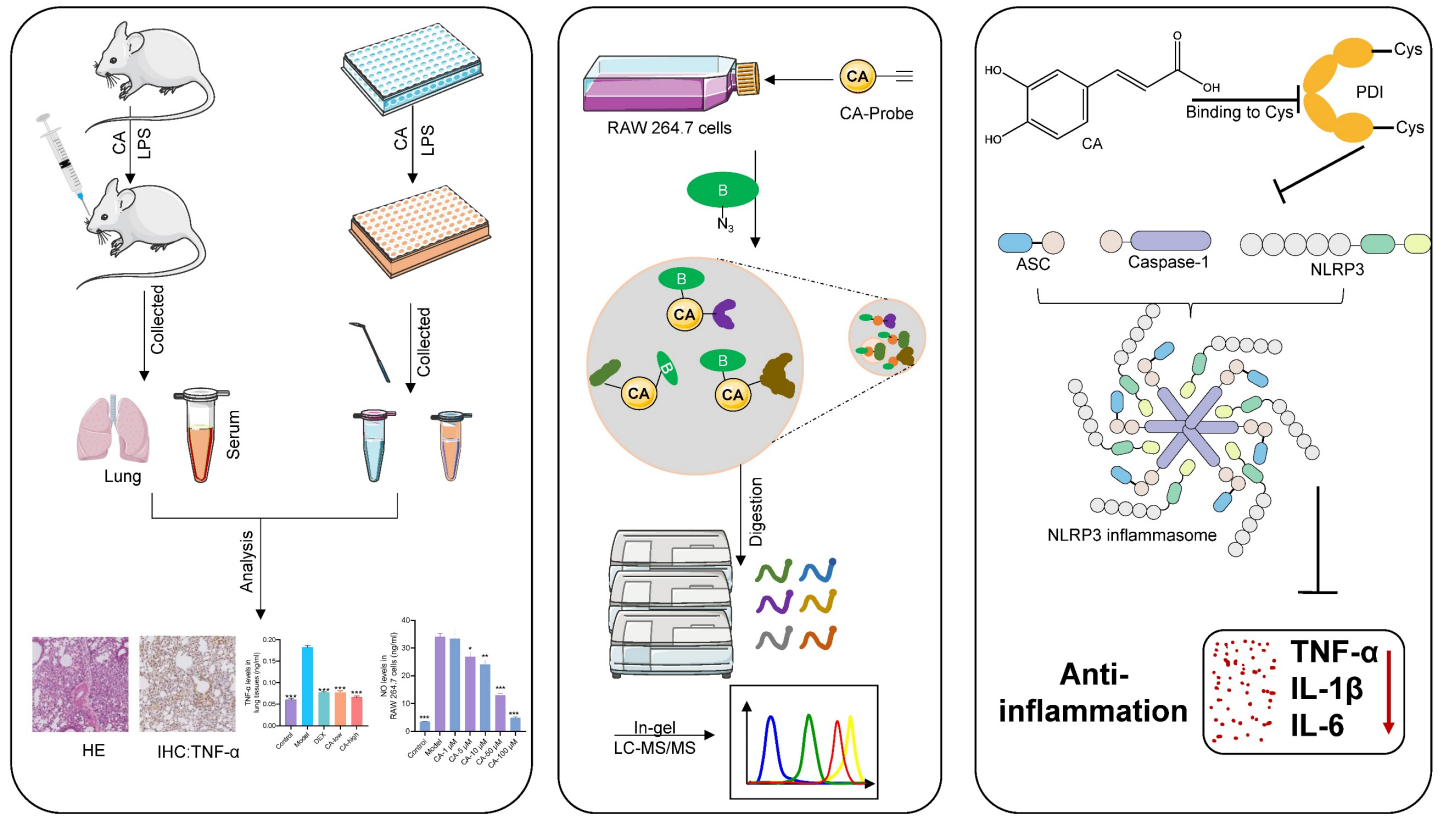
Application strategy to uncover potential targets and molecular pathways responsible for the anti-inflammatory properties of CA in acute pneumonia.

To the best of our knowledge, this study provides the first detailed report elucidating the observed anti-inflammatory properties in both acute pneumonia mouse models and LPS-induced macrophages. We believe that the data and insights obtained from this investigation could provide valuable guidance for the future clinical application of CA (**Figure 8**). While adverse effects associated with anti-inflammatory medications affect only a minority of patients, the widespread use of these drugs has led to a significant number of individuals experiencing serious side effects, such as gastrointestinal reactions with aspirin and metabolic disorders with glucocorticoids 29, 30. Therefore, there is an urgent need for the development of specialized anti-inflammatory agents derived from natural sources that provide both high efficacy and safety. Considering that coffee, a globally consumed beverage, is a primary source of CA 31, it is notable that research has highlighted the pivotal role of CA in numerous anti-inflammatory processes 7, 32. Our study unequivocally demonstrates a correlation between CA and anti-inflammatory effects in acute pneumonia through animal experiments, with high doses showing comparable effects to the conventional drug dexamethasone, thus highlighting the potential clinical utility of CA. Furthermore, consistent with these findings, our *in vitro* results also showed significant anti-inflammatory effects of CA in LPS-induced RAW264.7 cells. The remarkable anti-inflammatory activities of CA observed in both cellular and mouse models have inspired us to investigate the underlying mechanisms further.

Leveraging the high-throughput capabilities and compatibility with living systems provided by chemoproteomics, we identified the PDI family as a primary covalent target of CA. This discovery was substantiated by a comprehensive series of validations, including pull-down assays and immunofluorescence techniques. PDI, a redox-sensitive chaperone abundantly present in the endoplasmic reticulum (ER), is essential for facilitating proper disulfide bond formation 33. Furthermore, cysteine residues located at the catalytic center of PDI have been reported to participate in enzymatic processes 34. In alignment with this understanding, our investigations revealed that CA forms covalent bonds with the cysteine residues of PDI, which might result in a substantial inhibition of its reductive activity. Thus, PDI emerged as the functional target of CA responsible for its anti-inflammatory activity.

Previous studies have not successfully elucidated the specific molecular mechanism by which CA treats acute pneumonia. The NLRP3 inflammasome has been a major focus in the study of immune and inflammatory diseases 35. Our research offers new insights, as evidence from both *in vitro* and *in vivo* experiments suggests that the PDI-mediated NLRP3 inflammasome is the specific mechanism underlying CA's effectiveness in acute pneumonia. This discovery identifies a potential novel anti-inflammatory target and lays the groundwork for developing other anti-inflammatory natural medicines that target PDI. While our findings highlight the significance of the PDI-mediated NLRP3 inflammasome, we acknowledge that CA may also reduce NLRP3 inflammasome expression through other targets or affect other inflammatory pathways via PDI, contributing to its anti-inflammatory effects, although this requires further investigation.

We recognize several limitations in our study that require further investigation. Firstly, the natural drug CA may have multiple targets contributing to its anti-inflammatory effects, as indicated by the covalent binding of CA to other proteins identified in the MS data. Therefore, further investigation is needed to understand the roles of these additional protein targets in CA's anti-inflammatory mechanism. Additionally, considering the importance of the PDI family in inflammation highlighted in our study, it is crucial to investigate whether other proteins within the PDI family participate in CA-mediated anti-inflammatory pathways. Finally, although our study showed that CA covalently binds to cysteines within the catalytic center of PDI, the effect of this binding on the enzymatic activity of PDI is still unclear. Further research is needed to elucidate how CA affects the enzymatic activity of PDI and whether this aspect contributes to the anti-inflammatory activity of CA.

## Conclusion

In summary, our study marks the pioneering application of activity-based protein profiling (ABPP) in conjunction with bio-orthogonal click chemistry reaction and cellular thermal shift assay (CETSA) to elucidate the direct targeting of PDI by CA. Subsequent molecular biology investigations revealed that CA selectively covalently binds to the four cysteines of PDI, with the PDI mediated NLRP3 inflammasome pathway serving as the specific mechanism through which CA alleviates inflammation both *in vivo* and *in vitro*. These findings present new therapeutic avenues for treating acute pneumonia and offer fresh insights into the anti-inflammatory properties of CA.

## Supplementary Material

Supplementary figures and tables.

## Data Availability

All data analyzed within this study are included either in the manuscript or in the additional files.

## Abbreviations

ABPP: activity-based protein profiling
CA: caffeic acid
CA-P: caffeic acid probe
LPS: lipopolysaccharide
PDI: protein disulfide isomerase
IL-1: interleukin-1
IL-6: interleukin-6
TNF-α: tumor necrosis factor alpha
CETSA: cellular thermal shift assay
LC-MS/MS: liquid chromatography-tandem mass spectrometry
ELISA: enzyme-linked immune sorbent assay
