# Supplementary material for: Caffeic Acid Modulates Protein Disulfide Isomerase-NLRP3 Inflammasome Signaling to Mitigate Inflammation in Acute Pneumonia
Source: Int J Biol Sci. 2026 Mar 9;22(6):3288–303. doi: 10.7150/ijbs.101061 (PMC13050474; doi:10.7150/ijbs.101061)
Supplement: Supplementary file 1 — Supplementary figures and tables. [file ijbsv22p3288s1.zip › Supplementary materials.pdf]

Supplementary materials

Caffeic Acid Modulates Protein Disulfide Isomerase-NLRP3 Inflammasome Signaling to Mitigate Inflammation in Acute Pneumonia

Guanjun Li[1,2,3#], Tong Yang[1#], Ying Zhang[1#], Ang Ma[1], Lirun Zhou[1], Chen Wang[1], Peng Gao[1], Ang Ma[1], Cui Liu[1], Junzhe Zhang[1], Yin Hua Zhu[4*], Huan Tang[1*], Jigang Wang[1,3,5*]

[1] State Key Laboratory for Quality Ensurance and Sustainable Use of Dao-di Herbs, Artemisinin Research Center, and Institute of Chinese Materia Medica, China Academy of Chinese Medical Sciences, Beijing 100700, China.

[2] State Key Laboratory of Veterinary Public Health and Safety, College of Veterinary Medicine, China Agricultural University, Beijing 100193, China.

[3] Department of Urology, Shenzhen Clinical Research Centre for Geriatrics, Shenzhen People's Hospital; The First Affiliated Hospital, Southern University of Science and Technology, Shenzhen 518020, Guangdong, China.

[4] Beijing Advanced Innovation Center for Food Nutrition and Human Health, Department of Nutrition and Health, China Agricultural University, Beijing 100193, China.

[5] State Key Laboratory of Antiviral Drugs, School of Pharmacy, Henan University, Kaifeng, China.

[#] Guanjun Li, Tong Yang, and Ying Zhang contributed equally to this work.

* Corresponding Author:

Prof. Huan Tang, E-mail address: htang@icmm.ac.cn;

Prof. Yinhua Zhu, E-mail address: zhuyinhua@cau.edu.cn;

Prof. Jigang Wang, E-mail address: wangjigang@u.nus.edu.

Table legends.

Table S1. Raw data of LC-MS/MS.

Figure legends.

A

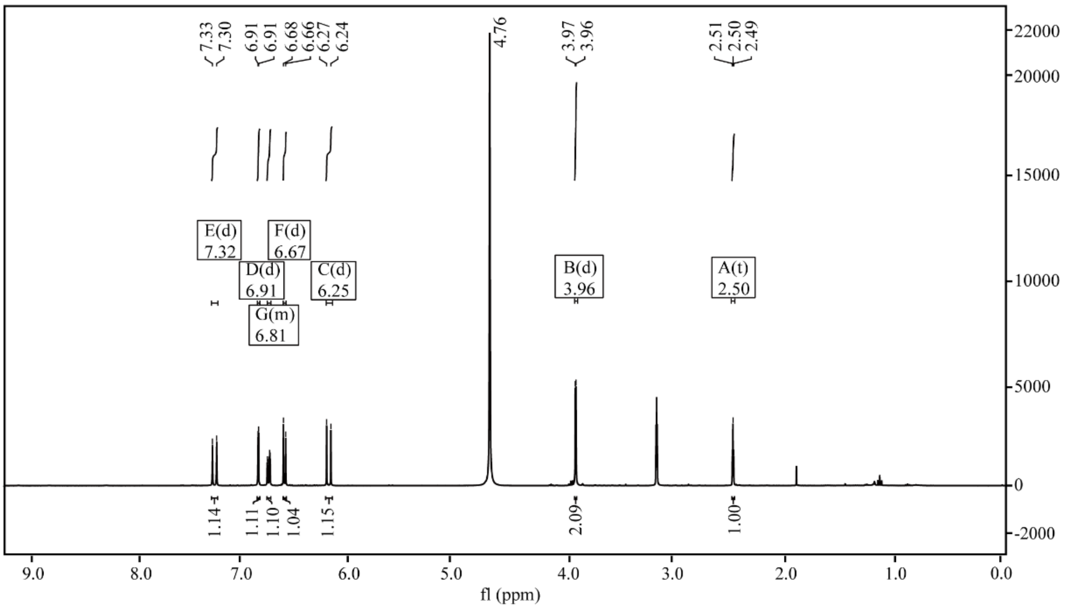

B

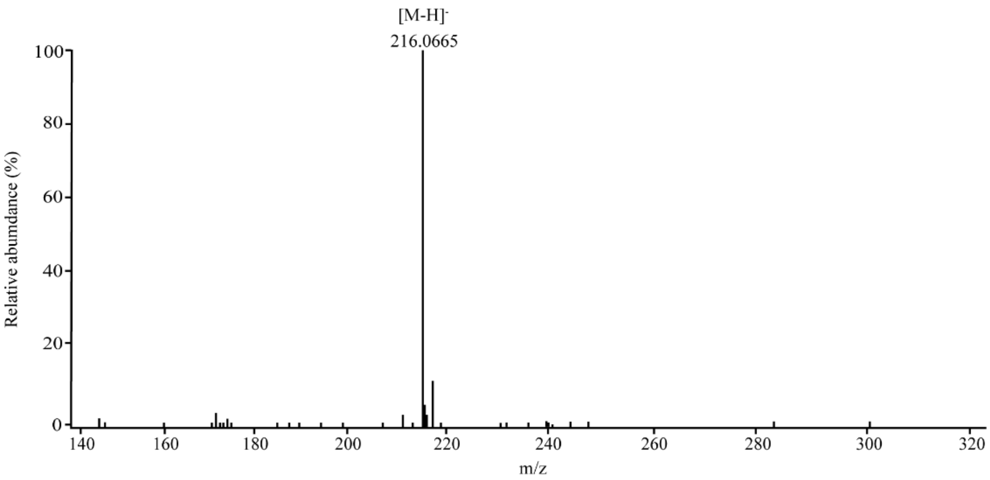

Figure S1. Characterization of CA-P. (A-B) The chemical structure of caffeic acid probe was characterized by H-NMR (A) and HRMS (B).

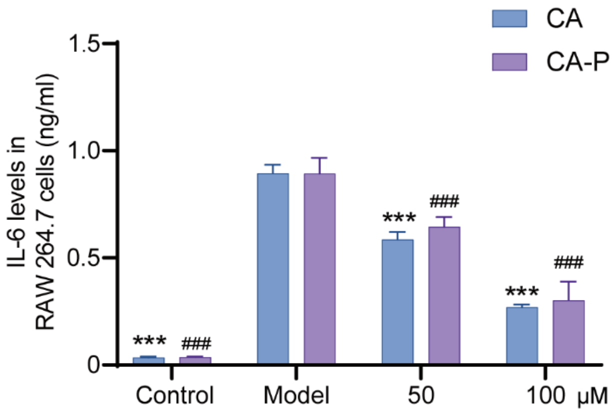

Figure S2. The levels of IL-6 in RAW 264.7 cells treated with CA or CA-P.

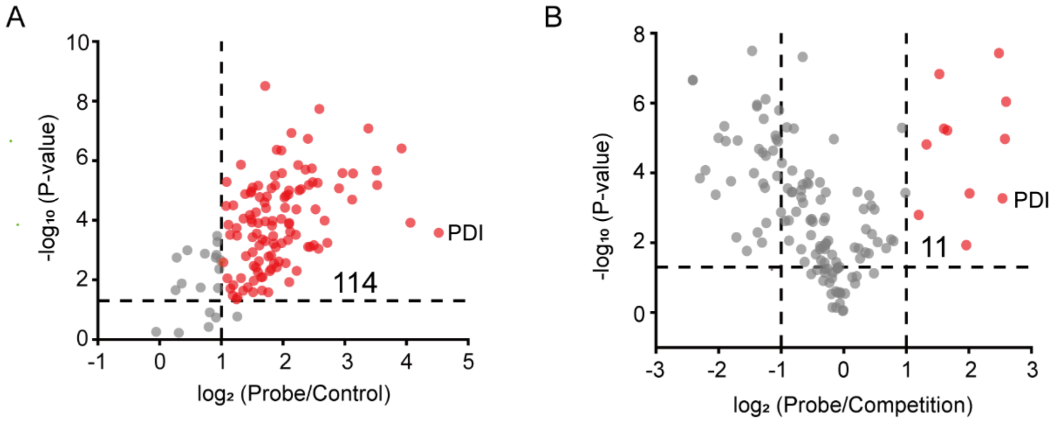

Figure S3. Volcano plot of the enriched proteins. (A-B) Volcano plot of the enriched protein in probe/control (A) and probe/competition (B). The P values

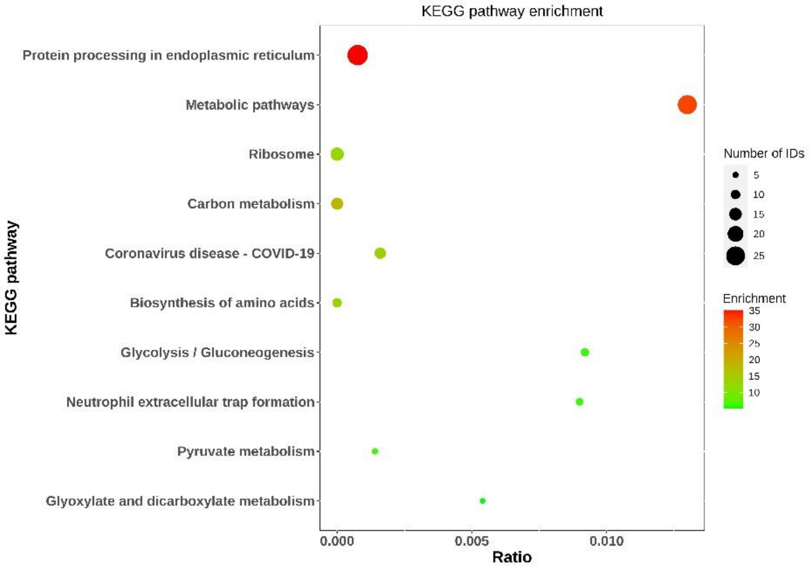

of the red points are less than 0.05.

Figure S4. KEGG pathway analysis of 114 protein targets identified in the probe/control group.

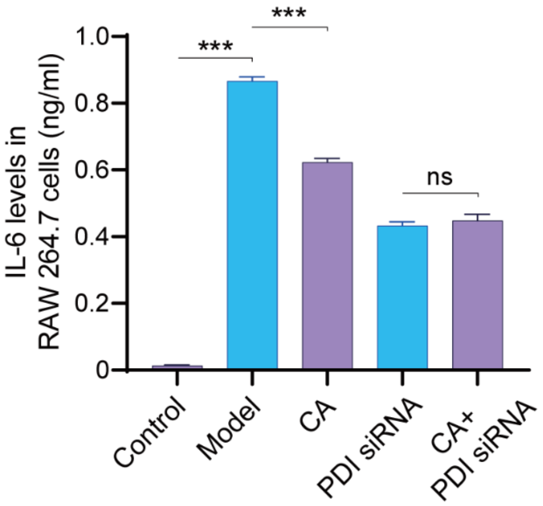

Figure S5. The levels of IL-6 in RAW 264.7 cells treated with CA and PDI siRNA.
